# Laryngeal Cancer in the West of Scotland 2014–2020: Trends and Survival in a Cohort of 867 Patients

**DOI:** 10.1002/lary.31992

**Published:** 2025-01-06

**Authors:** Rhona Hurley, Claire Paterson, David I. Conway, Gareth J. Inman, Catriona M. Douglas

**Affiliations:** ^1^ School of Cancer Sciences, Garscube Estate University of Glasgow Glasgow UK; ^2^ School of Medicine, Dentistry and Nursing University of Glasgow Glasgow UK; ^3^ Glasgow Head and Neck Cancer (GLAHNC) Research Group Glasgow UK; ^4^ Department of Otolaryngology/Head and Neck Surgery – Glasgow Royal infirmary and Queen Elizabeth University Hospital Glasgow UK; ^5^ Cancer Research UK Scotland Institute Glasgow UK; ^6^ Beatson West of Scotland Cancer Centre Glasgow UK

**Keywords:** cancer‐specific survival, laryngeal squamous cell cancer, overall survival

## Abstract

**Background:**

Laryngeal squamous cell cancer (LSCC) accounts for around one‐third of head and neck cancers, with smoking and alcohol as major risk factors. Despite advances in organ preservation, survival rates have stagnated globally over recent decades. The impact of socioeconomic deprivation on LSCC outcomes in the West of Scotland remains underexplored. We hypothesized that survival outcomes in the West of Scotland are poorer than cohorts from other developed nations.

**Aim:**

To evaluate characteristics and survival outcomes for LSCC patients in the West of Scotland and identify predictors of survival.

**Methods:**

A retrospective cohort study of 867 LSCC patients in the West of Scotland (2014–2020) analyzed demographics, tumor staging, performance status, treatments, and socioeconomic status (Scottish Index of Multiple Deprivation, SIMD). Subgroup differences were assessed using chi‐squared tests. Survival analysis was performed with Kaplan–Meier curves, log‐rank tests, and Cox proportional hazards modeling.

**Results:**

The cohort had a male‐to‐female ratio of 3.2:1, with a mean age of 65.5 years, with 56% presenting with advanced disease. Most patients (70.7%) lived in the most deprived areas. Supraglottic cancers were the most common subsite (51%). Five‐year overall survival (OS) was 46%, with a median OS of 52 months. Glottic cancers had better outcomes (64% OS) compared to supraglottic cancers (36%). Predictors of survival included age, subsite, performance status, alcohol use, treatment modality, and deprivation.

**Conclusion:**

LSCC survival in the West of Scotland is lower than in other European nations, influenced by advanced‐stage presentation, deprivation, and frailty. Addressing these factors is vital to improving outcomes.

**Level of Evidence:**

3 *Laryngoscope*, 135:2051–2061, 2025

## Graphical abstract

This retrospective cohort study of 867 patients with laryngeal squamous cell cancer (LSCC) in the West of Scotland (2014–2020) found a 5‐year overall survival rate of 46%, with glottic cancers having better outcomes (64%) compared to supraglottic cancers (36%). This study also found an increase in the presentation of supraglottic cancers over the study period.

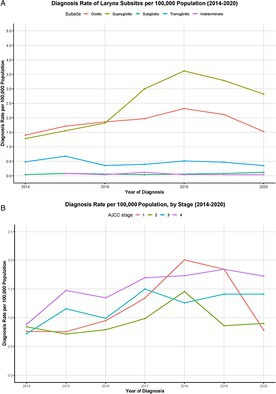

## INTRODUCTION

Larynx cancer represents one‐third of all head and neck cancers.^1^ Squamous cell cancer is the most prevalent histological type.^2^ Tobacco smoking and alcohol consumption are the predominant risk factors for the development of laryngeal squamous cell cancer.^3^ Treatment of laryngeal cancer is normally divided into treatment of early stage (T1/T2) and advanced stage (T3/T4) disease. Historically, early‐stage disease was treated with radiotherapy, but there has been a shift toward treating with transoral laser resection. Advanced stage disease was treated with laryngectomy but the landmark Veterans^4^ study heralded the shift toward organ preservation with chemoradiotherapy.

Five‐year survival of laryngeal cancer has historically been around 60%; however, recent publications have noted that despite the shift in management of laryngeal cancer, survival has not improved.^5^, ^6^ This is consistent across various European countries including Finland, Sweden,^7^ Denmark,^8^ and Ireland.^9^ Following the move to organ preservation in the form of radiation alone or chemoradiation, survival in laryngeal cancer was noted to decrease in the United States.^10^ Megwalu et al. demonstrated favorable survival outcomes for surgery compared with radiotherapy in early‐stage disease in a population‐based study in the United States.^11^

Laryngeal cancer incidence has been reported to be decreasing in the United Kingdom^12^ and the United States^13^ over the past 30 years, which may be related to decreasing trends in smoking. In Scotland, smoking rates have decreased from 28% of adults self‐reporting as current smokers in 2003 to 15% in 2022.^14^ A Danish study found that incidence of laryngeal cancer decreased overall between 1994 and 2018 but this varied by socioeconomic status (years of education), with a decrease in those with long/medium education and increase in those with short education.^15^ Despite a decrease in incidence rates of laryngeal cancer there has not been any reported change in survival outcomes with those diagnosed with the disease.^13^ This contrasts with progress in survival in other cancer types.^7^, ^16^, ^17^

The aim of this study is to describe the incidence and survival characteristics of a large cohort of laryngeal cancer patients treated in a cancer network in the United Kingdom.

## MATERIALS AND METHODS

This is a retrospective cohort study of patients diagnosed with laryngeal squamous cell cancer (LSCC) in the West of Scotland, United Kingdom, identified from the cancer network database from 2014 to 2020. The cancer network covers a population of around 2.5 million. Patients with carcinoma in situ diagnosed on primary biopsy pathology discussed at Multidisciplinary Tumour Board Meeting (MDT) were included if this progressed during their follow‐up, or if they had coexisting malignant SCC lymphadenopathy as it was assumed that invasive malignancy was not detected on primary biopsy.

Data on sociodemographics, tumor stage, treatment, American Society of Anesthesiologists (ASA) scores, and Eastern Cooperative Oncology Group (ECOG) performance status (PS) were captured from electronic case notes. Increasing‐risk alcohol use was defined as >21 units per week, as defined by UK National Institute for Health and Care Excellence.^18^ Staging data were based on American Joint Committee on Cancer (AJCC)/TNM 7 by MDT consensus as most patients were diagnosed prior to adoption of TNM 8 in our center in 2018 and no significant changes with regard to laryngeal cancer staging exist between TNM7 and TNM8.^19^ Some patients were not formally staged as they were managed with supportive care only.

The area‐based socioeconomic level of the patient's home address was determined using the Scottish Index of Multiple Deprivation (SIMD). This index is based on levels of income, employment, education, health, access to services, crime, and housing in the postcode area. Quintiles were used to indicate level of socioeconomic deprivation (1 being most deprived, 5 being least deprived).^20^

Population covered by MDT was calculated based on information on council area populations published by the Scottish Government from 2014 to 2020.^21^ The population covered by the West of Scotland MDT increased from 2,493,890 to 2,555,590 during this period (2.47% increase). The diagnosis rate per 100,000 population was then evaluated on a yearly basis and classified by site and stage. Both overall and cancer‐specific survival (CSS) were analyzed, measured from date of diagnosis, with treatment‐related death (i.e., aspiration after radiotherapy treatment, or blocked laryngectomy) being included as cancer‐related death.

Statistical analysis was performed in RStudio v4.3.1. Chi‐squared tests were used to evaluate differences between groups. Kaplan–Meier survival analysis with log‐rank testing was employed for univariate analysis, while Cox proportional hazards modeling was used for multivariate survival analysis. Survival was calculated from date of MDT diagnosis.

Caldicott approval and UK Research Ethics Committee approval was obtained (IRAS project ID 333481).

## RESULTS

### Patient Demographics

There were 867 patients identified who were diagnosed with laryngeal cancer in the West of Scotland between 2014 and 2020. Mean age at diagnosis was 65.5 years old (range 21–97).

Demographic distribution, SIMD quintiles, stage and subsite, smoking status, PS, ASA scores, and primary treatment modality are shown in Table I. Key findings are that 56% presented with advanced disease (AJCC stage III or IV) and that 70.7% live within the most deprived two quintiles.

**Table I:** Demographic, Staging, Comorbidity, and Treatment Information for the Entire Cohort of 867 Patients.

	Number of Patients and Percentage
Gender	660 male (76.1%)
	207 female (23.9%)
Age (years)	Under 60–244 (28.1%)
	60–70–335 (38.6%)
	Over 70–288 (33.2%)
Smoking status at time of diagnosis	Current – 504 (58.1%)
	Ex‐smoker – 289 (33.3%)
	Never smoked – 74 (8.5%)
Alcohol consumptionC†	Non‐alcohol drinker – 211 (24.3%)
	Hazardous alcohol drinking levels – 265 (30.7%)
	Occasional alcohol drinker – 280 (32.3%)
	Previous hazardous drinking levels – 75 (8.7%)
	Not recorded – 32 (3.6%)
Opioid use	Yes, or previous use – 54 (6.3%)
	No – (93.6%)
Substance misuse	Yes, or previous use – 71 (8.2%)
	No – 796 (91.8%)
AJCC staging	1–214 (24.7%)
	2–166 (19.1%)
	3–214 (24.6%)
	4–271 (31.3%)
	Not staged – 2 (0.2%)
T staging	Tcis – 4 (0.46%)
	T1–214 (24.7%)
	T2–190 (21.9%)
	T3–262 (30.2%)
	T4–195 (22.5%)
	Not staged – 2 (0.2%)
N staging	N0–653 (75.3%)
	N1–51 (5.9%)
	N2 (NOS)* – 8 (9.2%)
	N2a – 5 (0.6%)
	N2b – 62 (7.2%)
	N2c – 58 (6.6%)
	N3 (NOS)* – 4 (0.46%)
	N3a – 0 (0%)
	N3b – 24 (2.8%)
	Not staged – 2 (0.2%)
M staging	M0–854 (98.5%)
	M1–12 (1.4%)
	Not staged – 1 (0.1%)
Laryngeal subsite involvement	Supraglottis – 441 (51%)
	Glottis – 327 (37.7%)
	Subglottis – 9 (1.3%)
	Transglottic – 82 (9.4%)
	Indeterminate – 8 (0.9%)
WHO ECOG performance status	0–349 (40.3%)
	1–302 (34.8%)
	2–151 (17.4%)
	3–62 (7.2%)
	4–3 (0.3%)
ASA score	0–6 (0.7%)
	1–23 (2.7%)
	2–168 (19.7%)
	3–415 (48.7%)
	4–51 (6%)
	Missing data – 204 (23.9%)
SIMD quintile	1–411 (47.4%)
	2–202 (23.3%)
	3–105 (12.1)
	4–69 (8.0%)
	5–67 (7.7%)
	Missing data – 13 (1.5%)
Treatment intent	Curative – 663 (76.4%)
	Palliative – 204 (23.5%)
Primary treatment in curative group	Radiotherapy – 284 (42.8%)
	Total laryngectomy – 204 (30.8%)
	Chemoradiotherapy – 38 (5.7%)
	Transoral laser excision – 137 (20.6%)
	Partial laryngectomy – 3 (0.5%)
Summary of treatment modalities in patients treated with curative intent (*n* = 663) **‡**	Conservative surgery only – 132 (20%)
	Radical surgery only – 128 (19.3%)
	Radiotherapy only – 282 (42.5%)
	Conservative surgery and radiotherapy – 6 (0.9%)
	Radical surgery and radiotherapy – 70 (10.6%)
	Chemoradiotherapy – 36 (5.43%)
	Trimodality – 11 (1.66%)

Abbreviations: ASA, American Society of Anesthesiologists; ECOG, Eastern Cooperative Oncology Group; SIMD, Scottish Index of Multiple Deprivation.

* NOS—not otherwise specified.

^**†**^ Hazardous drinking defined as >21 units per week.

^**‡**^ — Conservative surgery” is defined as treatment with transoral laser (*n* = 131) and partial laryngectomy (*n* = 3). Conservative surgery and radiotherapy is treatment with conservative surgery as detailed above and subsequent radiotherapy treatment due to positive margins. Radical surgery is total laryngectomy +/− pharyngectomy +/− neck dissection and neck dissections. Trimodality encompasses radical surgery as defined above, and chemoradiotherapy.

Recurrence rate was 20.8% within the cohort (*n* = 180). Of these, 63.9% (115) were local recurrence, 25% (45) were regional, and 11.1% (20) were distant.

### Treatment of Laryngeal Cancer

Approximately 76.1% of patients were treated with curative intent, and 23.9% were treated with palliative intent. The most common treatment utilized was single modality radiotherapy (42.9%) followed by total laryngectomy (30.8%) (20.1%). Table I summarizes the treatment modalities for the entire cohort, including whether or not they had multimodality treatment in patients treated with curative intent. A more detailed comparison is provided in Table II, which examines treatment variations across subsites and disease stages. This indicates a highly statistically significant difference in treatment modalities based on laryngeal subsite involvement and disease stage. Glottic cancers and early‐stage disease appear to be more likely to be treated with single modality treatment.

**Table II:** Comparison of Treatment Modality by Subsite and Stage in Curatively Treated Laryngeal Cancer.

Treatment Modality	Subsite				
	Supraglottic (*n* = 303)	Glottic (*n* = 288)	Subglottic (*n* = 5)	Transglottic (*n* = 62)	Indeterminate (*n* = 5)
Conservative Surgery Only	29 (9.6%)	102 (35.4%)	0 (0%)	1 (1.6%)	0 (0%)
Conservative Surgery and Radiotherapy	1 (0.3%)	5 (1.7%)	0 (0%)	0 (0%)	0 (0%)
Radiotherapy Only	128 (42.6%)	142 (49.3%)	1 (20%)	10 (16.3%)	0 (0%)
Radical Surgery Only	74 (24.4%)	20 (6.9%)	3 (60%)	28 (45.2%)	3 (60%)
Radical Surgery and Postoperative Radiotherapy	35 (11.6%)	14 (4.86%)	1 (20%)	18 (29.0%)	1 (20%)
Chemoradiotherapy	28 (9.2%)	3 (1%)	0 (0%)	4 (6.5%)	1 (20%)
Trimodality	8 (2.6%)	2 (0.7%)	0 (0%)	1 (1.6%)	0 (0%)
	**Early stage (*n* = 347)**	**Advanced stage (*n* = 316)**	Chi sq. result for treatment modality versus subsite *p* < 0.001		
Conservative Surgery Only	125 (36.0%)	7 (2.2%)			
Conservative Surgery and Radiotherapy	5 (1.4%)	1 (0.3%)	Chi sq. result for treatment modality versus stage *p* < 0.001		
Radiotherapy Only	208 (59.9%)	74 (23.4%)			
Radical Surgery Only	9 (2.6%)	119 (37.7%)			
Radical Surgery and Postoperative Radiotherapy	0 (0%)	70 (22.1%)			
Chemoradiotherapy	0 (0%)	35 (11.1%)			
Trimodality	0 (0%)	10 (3.2%)			

*Note*: This table presents the comparison between treatment modalities by subsite and stage. Chi‐squared testing indicated a statistically significant relationship between site and treatment modality and stage and treatment modality.

Ninety‐three (14.6%) patients experienced recurrence after nonsurgical treatment. Fourteen recurrences occured after chemoradiotherapy and 79 after radiotherapy giving a recurrence rate of 38.9% and 28.0% for each treatment, respectively. Eight patients (57.1%) received salvage treatment after chemoradiotherapy comprising of seven salvage laryngectomies and one salvage neck dissection. Forty‐seven patients (59.4%) underwent salvage treatment after radiotherapy alone, consisting of 43 patients undergoing salvage laryngectomy, two patients undergoing salvage neck dissection, and one patient each undergoing salvage laser and salvage partial laryngectomy, respectively.

### Whole Cohort Survival Analysis

**Figure 1:**
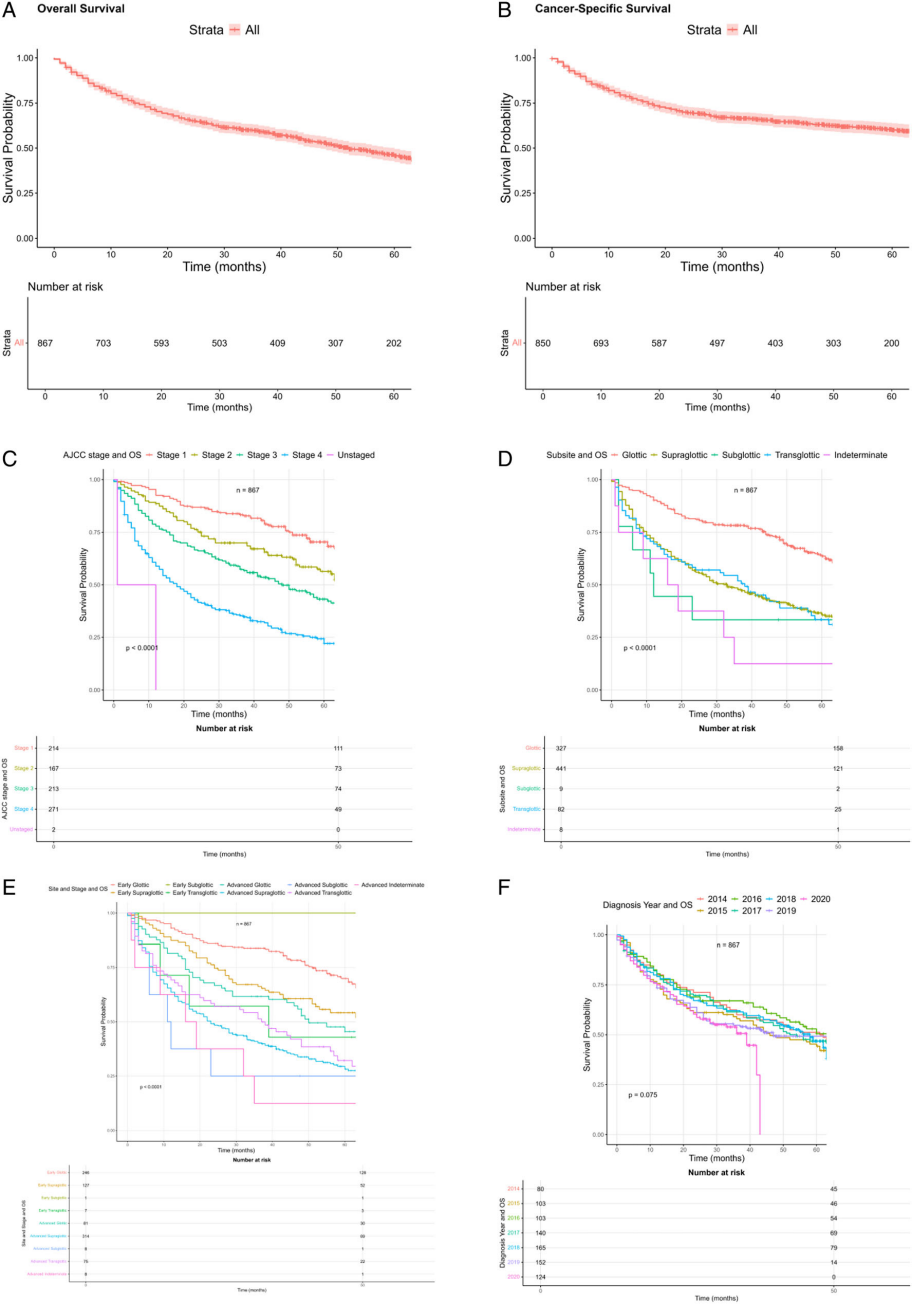
(A–F) Overall (A) and cancer‐specific (B) survival curves for the entire cohort. Five‐year survival analysis (*n* = 867) is performed with censoring of patients who did not have full 5‐year follow‐up. (C) Overall survival by laryngeal cancer AJCC stage in the whole cohort (*n* = 867). (D) Laryngeal cancer overall survival by subsite. Transglottic, supraglottic, and subglottic survival appears to be less favorable than survival in glottic cancers (*n* = 867). (E) Overall survival by stage and site in the whole cohort (*n* = 867). Here, the interaction between advanced disease and site shows a greater effect. (F) Kaplan–Meier curve showing overall survival in laryngeal cancer by year. (*n* = 867). [Color figure can be viewed in the online issue, which is available at www.laryngoscope.com.]

Overall survival (OS) by 5 years in the whole cohort was 46% and median survival was 52 months. Cancer‐specific survival by 5 years was 60% and median survival was 90 months. Overall and cancer‐specific survival curves for the whole cohort are shown in Figure 1A,B. For curative treatment only, 5‐year OS was 58.6% and median survival was 53 months. Five‐year CSS was 76.4%, median survival was 53.8 months. In those who experienced recurrence after nonsurgical treatment, 5‐year OS was 36% and median survival was 39 months; 5‐year CSS was 42.4% and median survival was 46 months.

### Overall Survival by Stage and Site

Kaplan–Meier curves demonstrating overall survival by AJCC staging is shown in Figure 1C for the whole cohort. As expected, increasing stage confers poorer survival. Overall survival by laryngeal subsite for the whole cohort is shown in Figure 1D. This demonstrates a more favorable survival outcome in glottic cancers (64% 5‐year OS, 77% 5‐year CSS), compared with other subsites (supraglottic 36% 5‐year OS, 52% CSS, subglottic 33% 5‐year OS and CSS, transglottic 33% 5‐year OS, 44.6% 5‐year CSS). Detailed results are shown in Table III. However, when combined as variables, it appears that stage is an important factor and advanced disease (T3/T4) for any site confers worse survival (Figure 1E). Tabulation of site and stage presentation and chi‐squared test results are shown in Table IV. This indicates that a higher proportion of glottic cancers present at an early stage.

**Table III:** Five‐Year Survival (OS and CSS) for all Subsites, Both by Whole Cohort and Curative Intent Treatment Group.

Five‐Year Survival by Subsite in Both Whole Cohort and Curative Intent Treatment Group				
Site	5‐year OS	95% CI	5‐year CSS	95% CI
Supraglottic – All	35.6%	30.9%–41.0%	51.6%	46.7%–57%
Supraglottic – Curative	51.3%	45.2%–58.2%	73.9%	68.4%–79.9%
Glottic – All	63.7%	58.1%–69.7%	76.9%	71.9%–82.1%
Glottic – Curative	70.8%	65.1%–77.0%	84.6%	79.8%–89.6%
Subglottic – All	33%	13.2%–8%	33%	46.7%–57%
Subglottic – Curative	60%	29.3%–100%	60%	29.3%–100%
Transglottic – All	33.4%	23.9%–46.6%	44.7%	33.8%–58.8%
Transglottic – Curative	43.1%	31.6%–59.0%	58.1%	45.5%–74.1%
Indeterminate – All	12.5%	2%–78.2%	18.7%	3.6%–97.6%
Indeterminate – Curative	20%	3.4%–100%	30%	6.3%–100%

**Table IV:** Chi‐squared Testing of Stage by Site. This Shows the Statistically Significant Differences Between Stage and Site, Indicating That a Higher Proportion of Patients Present With Supraglottic/Transglottic/Indeterminate/Subglottic Disease at an Advanced Stage Than Glottic.

Subsite	Early	Advanced	*p*‐value
Glottic	245 (Observed)	82 (Observed)	1.988187e‐19
	163.5 (Expected)	163.5 (Expected)	
Supraglottic	127 (Observed)	314 (Observed)	5.350075e‐19
	220.5 (Expected)	220.5 (Expected)	
Subglottic	1 (Observed)	8 (Observed)	1.963066e‐02
	4.5 (Expected)	4.5 (Expected)	
Transglottic	7 (Observed)	75 (Observed)	5.942449e‐14
	41 (Expected)	41 (Expected)	
Indeterminate	0 (Observed)	8 (Observed)	4.677735e‐03
	4 (Expected)	4 (Expected)	

### Temporal Trends in Diagnosis

**Figure 2:**
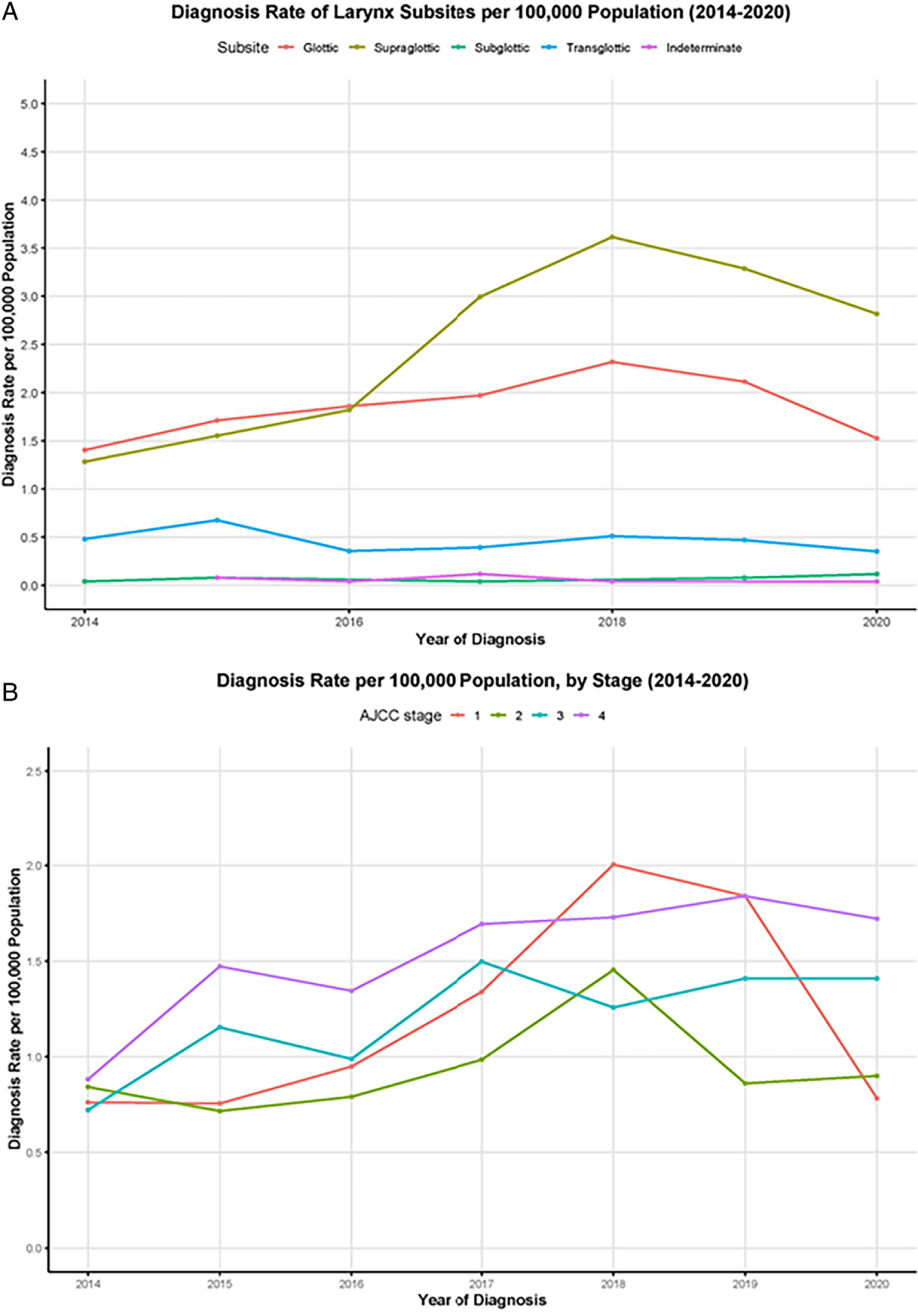
(A). Diagnosis trends by laryngeal subsite per 100,000 population. There appears to be an increase in diagnosis of supraglottic and glottic cancers from 2014 to 2018 followed by a decrease in rate to 2020. In particular, supraglottic cancer rates rose from 1.28 per 100,000 to a peak of 3.65 per 100,000 in 2018. (B) Diagnosis trends by stage per year—this shows an increase in Stage 4 disease over the time period (from 0.88 per 100,000 to 1.72 per 100,000), and an initial increase in Stage 1 disease before a decline in 2019 onward. [Color figure can be viewed in the online issue, which is available at www.laryngoscope.com.]

An increase in rate of diagnosis per 100,000 population of both glottic and supraglottic SCC was demonstrated until 2018, followed by a decline until the end of the study period in 2020. In particular, supraglottic cancer rates rose from 1.28 per 100,000 to a peak of 3.65 per 100,000 in 2018, remaining at 2.8 per 100,000 in 2020 (Figure 2A). There were low numbers overall diagnosed in 2014 and this is presumed to be a data recording issue.

When staging was examined, diagnosis of all stages of disease rose until 2018, with the most marked rises seen in stage 1 and stage 4 disease (Figure 2B). Stage 4 and Stage 3 disease diagnosis rates have remained relatively constant since 2018, whereas both stage 1 and stage 2 rates declined between 2018 and 2020 (Figure 2B).

Chi‐squared testing was used to determine the observed/expected counts for early and advanced stage disease. The results are shown in Table V, showing a greater than expected number of late‐stage disease in 2015, 2017, and 2020 (*p* = 0.025).

**Table V:** Contingency Table From Chi‐squared Test Showing Observed and Expected Counts Per Year. More patients than would be expected were diagnosed with advanced stage disease in 2015, 2017, and 2020. (*p* = 0.025, df = 6).

Contingency Table of Observed Versus Expected Cases by Year and Stage				
Year of Diagnosis		Disease Stage		
		Early	Advanced	Total
2014	Observed	40	40	80
	Expected	35.1	44.9	80.0
2015	Observed	37	66	103
	Expected	45.1	57.9	103.0
2016	Observed	44	59	103
	Expected	45.1	57.9	103.0
2017	Observed	59	81	140
	Expected	61.4	78.6	140.0
2018	Observed	88	77	165
	Expected	72.3	92.7	165.0
2019	Observed	69	83	152
	Expected	66.6	85.4	152.0
2020	Observed	43	81	124
	Expected	54.3	69.7	124.0
Total	Observed	380	487	867
	Expected	380.0	487.0	867.0

### Survival by Time and Stage at Presentation by Year and Relationship Between Comorbidities Over Time

A general trend of decreasing overall survival was found between 2014 and 2020, but this was only statistically significant in 2020 on univariate analysis (Table VI). To assess possible associations with this trend, linear regression analysis was conducted to assess the relationship between PS and the year of diagnosis, and age and year of diagnosis. The analysis revealed a statistically significant positive relationship between PS and diagnosis year (*p* = 0.036) indicating that PS tended to increase with increasing year. There was no statistically significant difference in age with year of diagnosis.

**Table VI:** Overall Survival Analysis in Curative Intent Treatment Group.

Variable	HR (Univariate)	HR Multivariate
Age Category		
Under 60	Ref	Ref
60–70	1.51 (1.13–2.04, *p* = 0.006*)	1.71 (1.23–2.39, *p* = 0.001*)
Over 70	2.06 (1.51–2.82, *p* < 0.001**)	2.66 (1.78–3.96, *p* < 0.001**)
Sex (ref: Female)	1.06 (0.80–1.42, *p* = 0.67)	‐
Smoking		
Nonsmoker	Ref	Ref
Current smoker	1.59 (1.01–2.50, *p* = 0.04*)	1.52 (0.91–2.56, *p* = 0.11)
Ex‐smoker	1.38 (0.86–2.23, *p* = 0.17)	1.17 (0.70–1.95, *p* = 0.54)
Alcohol		
Nondrinker	Ref	Ref
Occasional drinker	1.36 (0.97–1.92, *p* = 0.07)	1.53 (1.08–2.19, *p* = 0.02*)
Hazardous drinking	1.56 (1.10–2.18, *p* = 0.01*)	1.46 (1.01–2.11, *p* = 0.04*)
Previous hazardous drinking	2.15 (1.35–3.42, *p* = 0.001*)	2.26 (1.38–3.70 *p* = 0.001*)
Performance Status		
0	Ref	Ref
1	2.17 (1.68–2.81, *p* < 0.001**)	1.92 (1.45–2.53, *p* < 0.001**)
2	3.06 (2.18–4.29, *p* < 0.001**)	2.05 (1.41–3.00, *p* < 0.001**)
3	2.73 (1.00–7.42, *p* = 0.05)	3.67 (1.24–10.90, *p* = 0.02*)
Site		
Glottic	Ref	Ref
Supraglottic	1.85 (1.43–2.39, *p* < 0.001**)	1.57 (1.16–2.11, *p* = 0.002*)
Subglottic	1.70 (0.54–5.40, *p* = 0.36)	0.73 (0.09–5.55, *p* = 0.77)
Transglottic	2.06 (1.40–3.01, *p* < 0.001**)	1.51 (0.95–2.42, *p* = 0.08)
Indeterminate	5.47 (2.21–13.42, *p* < 0.001**)	5.73 (1.91–17.17, *p* = 0.001*)
Treatment		
Conservative Surgery Only	Ref	Ref
Radiotherapy Only	1.57 (1.09–2.27, *p* = 0.01*)	1.47 (0.97–2.20, *p* = 0.06)
Radical Surgery Only	2.06 (1.36–3.10, *p* < 0.001**)	1.06 (0.62–1.82, *p* = 0.83)
Conservative Surgery and Postoperative Radiotherapy	0.29 (0.04–2.19, *p* = 0.23)	0.31 (0.04–2.32, *p* = 0.26)
Radical Surgery and Postoperative Radiotherapy	2.43 (1.55–3.79, *p* < 0.001**)	1.73 (0.98–3.08, *p* = 0.06)
Chemoradiotherapy	1.84 (1.05–3.24, *p* = 0.03*)	1.48 (0.74–2.94, *p* = 0.25)
Trimodality	5.09 (2.52–10.28, *p* < 0.001**)	3.55 (1.55–8.15, *p* = 0.002*)
Diagnosis Year		
2014	Ref	Ref
2015	1.36 (0.89–2.06, *p* = 0.15)	1.07 (0.68–1.68, *p* = 0.75)
2016	1.02 (0.65–1.61, *p* = 0.93)	0.86 (0.52–1.40, *p* = 0.54)
2017	1.14 (0.73–1.78, *p* = 0.56)	0.86 (0.53–1.40, *p* = 0.54)
2018	1.09 (0.69–1.73, *p* = 0.7)	0.91 (0.56–1.48, *p* = 0.71)
2019	1.48 (0.93–2.34, *p* = 0.09)	1.21 (0.72–2.02, 0.46)
2020	1.83 (1.1–3.1, *p* = 0.02)*	1.50 (0.85–2.66, *p* = 0.16)
Early/Advanced Disease		
Early	Ref	Ref
Advanced	2.10 (1.65–2.66, *p* < 0.001)**	1.47 (1.02–2.12, *p* = 0.037*)
SIMD Quintile		
1	Ref	Ref
2	1.43 (1.07–1.9, *p* = 0.01)*	1.37 (1.00–1.88, 0.046)*
3	1.19 (0.81–1.74, *p* = 0.37)	1.21 (0.80–1.85, *p* = 0.36)
4	1.06 (0.70–1.62, *p* = 0.76)	1.12 (0.72–1.78, *p* = 0.60)
5	0.73 (0.44–1.19, *p* = 0.21)	0.64 (0.38–1.11, *p* = 0.12)
Not found	1.07 (0.40–2.92, *p* = 0.88)	1.01 (0.31–3.30 *p* = 0.99)

*Note*: Univariate and multivariate overall survival analyses, for the curative intent treatment group (*n* = 663). This reports hazard ratios, 95% confidence intervals, and *p*‐values. Advancing age, performance status, alcohol consumption, and disease stage confer worse survival on multivariate analysis. Also, supraglottic and indeterminate subsites, treatment with trimodality treatment are associated with statistically significant worse overall survival.

* *p* < 0.05.

** *p* < 0.001.

### Multivariate Overall Survival Analysis in Patients Treated With Curative Intent

Univariate survival analysis was performed to examine overall survival by age, sex, alcohol consumption, smoking status, performance status, stage, site, year of diagnosis, socioeconomic deprivation, and treatment modality in the curative treatment group (Table VI). Those factors which were significant on univariate analysis were included in multivariate analysis(Table VI). In summary, on multivariate analysis age, supraglottic/indeterminate subsites, SIMD quintile 2, performance status 1/2, alcohol consumption, treatment with trimodality treatment, and advanced disease confer significantly worse overall survival outcomes.

## DISCUSSION

This study reports a cohort of patients with laryngeal squamous cell cancer diagnosed between 2014 and 2020 from the West of Scotland, United Kingdom. The key findings are the survival outcomes of 5‐year OS 46%, 5‐year CSS 60% for all patients. In curative patients the 5‐year OS was 58.6% and 5‐year CSS was 76.4%. The majority (56%) of patients presented with advanced stage disease, and supraglottic cancer was the predominant subsite (*n* = 441, 51%). Lastly, performance status in this cohort appears to play a role in poor survival outcomes.

Our results suggest that both overall and cancer‐specific survival in our cohort of patients with laryngeal SCC is worse than similar cohorts, comparing unfavorably with Sweden (5‐year OS 65%),^22^ Norway (56.8% 5‐year OS, 80.2% 5‐year CSS)^23^, and Finland (63% OS in curative treatment, 80% CSS in curative treatment).^24^ An important factor could be the large proportion of patients (56%) presenting with advanced disease, which appears to be higher than other populations including Sweden(31.6%), Norway(39.2%), Finland(39%),^24^ the United States (46%),^25^ and England (30%–47.6%).^26^, ^27^. It is concerning that compared to previous reports of the Scottish population published 25 years ago, presentation with advanced disease has increased (56% now vs. 44%^26^).

The reasons for this tendency to presentation with advanced disease could include a lack of public awareness to consult with insidious symptoms which occur in non‐glottic cancer (in particular, supraglottic cancers) and/or the high proportion of patients affected by socioeconomic deprivation, which has previously been described to affect stage of presentation in laryngeal cancer.^28^ Scottish government statistics indicate a slight increase in poverty in working age adults since 1994 (rising from 18% to 21%),^29^ the age group most likely to be affected by laryngeal cancer. Knowledge of head and neck cancer and its presenting symptoms has been shown to be poor, particularly regarding laryngeal cancer,^30^ and knowledge is known to improve health‐seeking behavior.^31^ Furthermore, access to GP consultations remains a concern for many patients nationally,^32^, ^33^ and access is poorer for those from areas of high socioeconomic deprivation.^34^

A previous study from our center indicates an association between recreational drug misuse and supraglottic cancer^35^ and we note 8.2% incidence of current or prior drug misuse in our cohort. This is possibly an underestimate as this was recorded from the electronic records, which would require enquiry regarding drug use and documentation by clinicians. The Scottish Crime and Justice Survey 2018–2020 noted that 10% of survey respondents self‐reported recreational drug misuse, with higher rates in areas of socioeconomic deprivation, increasing to 15%.^36^ It is notable that Scotland has the highest rate of drug‐related death in Europe, with the majority associated with opiate misuse and deprivation.^37^ This association with drug misuse has been noted in an Israeli cohort in which supraglottic SCC in intravenous drug users (IVDU) was compared with supraglottic cancers in non‐IVDU. Supraglottic SCC which occurred in the IV drug users were found to present at a younger age and have improved overall survival, despite similar comorbidity status between the two groups. IVDU was protective in multivariate survival analysis.^38^ This raises the question of disease phenotype in this cohort and certainly the effect of recreational drug use in laryngeal cancer warrants further investigation.

In our study 59.7% of patients had a PS of 1 or more and increasing PS was found to be associated with worse survival. Poor PS (1, 2) has also been shown to influence progression‐free survival in HNSCC.^39^ Performance status has been criticized for its subjectivity, which may lead to interobserver variability between assessments and subsequent impact on decision‐making and outcomes.^40^, ^41^ There has been evidence that other frailty measures may be useful predictors of outcomes in HNSCC.^42^

We note that 39.7% of patients in our cohort consumed alcohol at a hazardous level (>21 units per week) or had previously done so. In multivariate analysis, consumption of alcohol at any level is associated with statistically significant poorer OS. This is in keeping with recent research indicating that even moderate alcohol consumption of 10–20 units per week confers poorer overall survival in HNSCC,^43^, ^44^ highlighting the importance of supporting patients in reducing their alcohol intake. A key focus in head and neck cancer patients after diagnosis is often support for smoking cessation, given the long‐standing strong evidence^45^ to show that this reduces the chance of recurrence. However, there should also be a focus toward supporting patients who drink to excess giving the emerging evidence that alcohol plays in survival outcomes in HNSCC.

In line with other studies, glottic cancer confers better survival than other cancer types. This contrast is particularly stark due to the proportion of supraglottic cancers in this cohort. Glottic cancer is likely to have favorable outcomes due to its tendency to present with dysphonia allowing earlier diagnosis and treatment before disease reaches an advanced stage in almost 75% of cases, as well as lack of propensity for lymph node metastasis. The predominance of supraglottic cancers in this cohort will have an impact on presentation stage due to their tendency to present with unilateral throat pain and otalgia, as opposed to dysphonia. This is likely to contribute to the high proportion of advanced disease in this cohort. Other subsites are more likely to present at advanced stage (Table IV) and this is reflected in poor survival outcomes when compared with glottic cancers (Table III), with a 11% difference in cancer‐specific survival between glottic and supraglottic cancers in patients treated with curative intent, and 25% difference in cancer‐specific survival in the whole cohort.

The profile of laryngeal cancer has changed in this cohort, as supraglottic cancers are the most common subsite in our cohort, which contrasts with other datasets.^9^, ^13^ Previous reports of Scotland‐wide data from 1999 to 2001 described the proportion of supraglottic cancers as 34.8% (personal communication of unpublished data, available on request). This change could explain the lack of improvement in overall survival of the whole cohort over the study period and warrants further investigation to understand the potential causes of this. The better outcomes in IVDU with supraglottic cancer^38^ as well as previous analysis of data from The Cancer Genome Atlas have shown different subsite LSCCs with different molecular profiles^46^ may indicate that there is an unknown causative factor (e.g., virus or immunosuppression) which may play a role in supraglottic cancer.^46^ To our knowledge, this is the first publication to explicitly demonstrate increase in supraglottic subsite laryngeal cancer from the period 2015–2020 and is in contrast to a recent large study that indicated no such change in US population‐based data.^13^

Approximately 70.7% of the patients in this cohort live in areas of high socioeconomic deprivation, which has been shown to be associated with late stage at presentation.^28^ However, previous analysis of Scottish head and neck cancer data found that the effect of socioeconomic deprivation on long‐term cause specific survival was not found to be significant after adjustment for patient factors and tumor/treatment variables.^47^ In our study, patients in SIMD quintile 2 appear to have worse survival outcomes on univariate analysis compared with SIMD quintile 1, which is the most deprived, reinforcing that the relationship between deprivation and laryngeal cancer survival is not simple. This underpins the complexity in understanding worsening survival in LSCC which is likely to be multifactorial in nature.

Approximately 63% of patients in our cohort with advanced disease receive only single modality treatment, likely a reflection of poor performance status; 60% of patients in this cohort had a performance status of 1 or above. Glasgow, the population center in the West of Scotland, is known for its 30% excess premature deaths in comparison to other cities with similar populations and similar levels of deprivation in England. Half of these are related to alcohol or drugs.^48^ Therefore, treatment decisions in this cohort are driven by poor health status and comorbidities dictating the choice of single modality treatment and in turn, poor outcomes which are of course also influenced by poor health and comorbidities in this cohort.

Only a small proportion of patients in this cohort received chemoradiotherapy (5.8%), likely due to poor performance status, comorbidities, and age. Evidence in the literature suggests that outcomes after radiotherapy alone in T3 disease are inferior to chemoradiotherapy or primary surgery.^49^ As nearly one‐quarter of patients with advanced disease received radiotherapy alone, this is likely to have an impact on our survival outcomes. Previous meta‐analysis^50^ has shown benefit from concomitant chemotherapy in addition to radiotherapy or surgery in all locally advanced disease; however, this is not a universal finding and more recent meta‐analysis^51^ has found that radiotherapy alone conferred best survival outcomes in locally advanced disease. Furthermore, addition of chemotherapy to radiotherapy has less benefit as age and performance status increase.^52^ Unfortunately, all treatments in advanced laryngeal cancer are highly morbid and this is an important consideration in treatment choice.

Limitations of this study include its retrospective nature and reliance on electronic patient records to gather information. Patients were defined as ex‐smokers or previous hazardous drinkers at the time of referral, and this does not reflect a specific minimum cessation interval as date of cessation of alcohol drinking or smoking was not known. TNM staging was taken from the cancer network record rather than divided into clinical, pathological, and radiological staging and this additional information could provide further details to the results. Cancer‐specific survival was defined as having a death related to laryngeal cancer. This was gleaned from death certificates if available, or if the patient's most recent clinic letters indicated the patient was for end‐of‐life care but was not available in all patients. SIMD quintiles are associated with limitations as they are an area‐based indicator, therefore, assuming that each group of 20% are homogenous at the individual, social, and economic level. Lastly, it would be useful to look in further depth at treatment outcomes separately between early and advanced disease.

## CONCLUSION

The lack of improved survival in laryngeal cancer over the last two decades, despite the advances in treatment, is alarming. This cohort compares unfavorably to data from similar countries within Europe, with subsite, alcohol consumption, advanced stage at presentation, and deprivation likely contributing factors to this. Supraglottic cancers appear to be the predominant subsite in our cohort, however, it is unclear what the social and/or biological basis/drivers for this may be. Translational research to better understand the biology of supraglottic disease should be a key area of focus moving forward.

## CONFLICT OF INTEREST STATEMENT

The authors report no conflicts of interest.

## Data Availability

Data can be made available upon reasonable request, with associated code for statistical analysis.

## References

[1] Society for Medical Oncology E . ESMO essentials for clinicians head and neck cancers. Essentials for Clinicians: Head and Neck Tumours and Neuro‐Oncology. Vol 1. Elsevier; 2017:1.

[2] Kawano T , Hirano T , Tateyama K , Yoshinaga K , Shinomura K , Suzuki M . Prognostic value of pretreatment inflammatory biomarkers in patients with laryngeal cancer. Asian J Surg. 2024;47:2144‐2151. 38311505 10.1016/j.asjsur.2024.01.073

[3] Khoueir N , Matar N , Farah C , et al. Survival of T4aN0 and T3N + laryngeal cancer patients: a retrospective institutional study and systematic review. American Journal of Otolaryngology ‐ Head and Neck Medicine and Surgery. W.B. Saunders; 2015:755‐762. 10.1016/j.amjoto.2015.07.009 26545467

[4] The Department of Veterans Affairs Laryngeal Cancer Study Group . Induction chemotherapy plus radiation compared with surgery plus radiation in patients with advanced laryngeal cancer. N Engl J Med. 1991;324:1685‐1690. 2034244 10.1056/NEJM199106133242402

[5] Timmermans AJ , Van Dijk BAC , Overbeek LIH , et al. Trends in treatment and survival for advanced laryngeal cancer: a 20‐year population‐based study in The Netherlands. Head and Neck. Vol 38. John Wiley and Sons Inc.; 2016:E1247‐E1255. 26315454 10.1002/hed.24200

[6] Francis E , Matar N , Khoueir N , Nassif C , Farah C , Haddad A . T4a laryngeal cancer survival: retrospective institutional analysis and systematic review. Laryngoscope. Vol 124. John Wiley and Sons Inc.; 2014:1618‐1623. 24338374 10.1002/lary.24557

[7] Koskinen A , Hemminki O , Försti A , Hemminki K . Incidence and survival in laryngeal and lung cancers in Finland and Sweden through a half century. PLoS One. 2022;17:e0268922. 35622857 10.1371/journal.pone.0268922 PMC9140270

[8] Nahavandipour A , Jakobsen KK , Grønhøj C , et al. Incidence and survival of laryngeal cancer in Denmark: a nation‐wide study from 1980 to 2014. Acta Oncol (Madr). 2019;58:977‐982. 10.1080/0284186X.2019.1572923 30821560

[9] Sexton GP , Walsh P , Moriarty F , Lennon P , O'Neill JP . Survival in an era of organ preservation: an update on laryngeal cancer in Ireland. Eur Arch Otorhinolaryngol. 2023;280:4587‐4595. 37326667 10.1007/s00405-023-08055-0 PMC10477096

[10] Hoffman HT , Porter K , Karnell LH , et al. Laryngeal cancer in the United States: changes in demographics, patterns of care, and survival. Laryngoscope. 2006;116:1‐13. 10.1097/01.mlg.0000236095.97947.26 16946667

[11] Megwalu U , Panossian H . Survival outcomes in early stage laryngeal cancer. Anticancer Res. 2016;36:2903‐2908. 27272804

[12] Shelton J , Zotow E , Smith L , et al. 25 year trends in cancer incidence and mortality among adults aged 35‐69 years in the UK, 1993‐2018: retrospective secondary analysis. BMJ. 2024;384:e076962. 38479774 10.1136/bmj-2023-076962 PMC10935512

[13] Divakar P , Davies L . Trends in incidence and mortality of larynx cancer in the US. JAMA Otolaryngol Head Neck Surg. 2022;1:34‐41. 10.1001/jamaoto.2022.3636 PMC9673027 36394832

[14] Hinchliffe S , Wilson V , et al. The Scottish Health Survey A National Statistics Publication for Scotland.2021.

[15] de la Cour CD , Munk C , Aalborg GL , Kjaer SK . Base of tongue/tonsillar and laryngeal cancer in Denmark 1994–2018: temporal trends in incidence according to education and age. Oral Oncol. 2022;128:105832. 35413640 10.1016/j.oraloncology.2022.105832

[16] Kim M.M. , Hoffman K.E. , Levy L.B. , *et al*. (2012) Improvement in prostate cancer survival over time a 20‐year analysis. 10.1097/PPO.0b013e3182467419 22290249

[17] Sundquist M , Brudin L , Tejler G . Improved survival in metastatic breast cancer 1985–2016. The Breast. 2017;31:46‐50. 27810699 10.1016/j.breast.2016.10.005

[18] disorders A. & harmful drinking Evidence Update March preventing. Alcohol‐Use Disorders: Preventing Harmful Drinking. NICE. 2014.

[19] Zanoni DK , Patel SG , Shah JP . Changes in the 8th edition of the American Joint Committee on Cancer (AJCC) staging of head and neck cancer: rationale and implications. Curr Oncol Rep. 2019;21:1‐22. 10.1007/s11912-019-0799-x PMC6528815 30997577

[20] Scottish Government . Scottish Index of Multiple Deprivation ‐ gov.scot. 2020.

[21] National Records of Scotland . Council Area Profiles. 2023.

[22] Blomkvist R , Marklund L , Hammarstedt‐Nordenvall L , Gottlieb‐Vedi E , Mäkitie A , Palmgren B . Treatment and outcome among patients with laryngeal squamous cell carcinoma in Stockholm—a population‐based study. Laryngoscope Investig Otolaryngol. 2023;8:441‐449. 10.1002/lio2.1034 PMC10116984 37090883

[23] Brandstorp‐Boesen J , Falk RS , Boysen M , et al. Impact of stage, management and recurrence on survival rates in laryngeal cancer. PLoS One. 2017;12:e0179371. 28708883 10.1371/journal.pone.0179371 PMC5510803

[24] Haapaniemi A , Koivunen P , Saarilahti K , et al. Laryngeal cancer in Finland: a 5‐year follow‐up study of 366 patients. Head Neck. 2016;38:36‐43. 24996171 10.1002/hed.23834

[25] Lebo NL , Khalil D , Balram A , et al. Influence of socioeconomic status on stage at presentation of laryngeal cancer in the United States. Otolaryngol Head Neck Surg. 2019;161:800‐806. 31184265 10.1177/0194599819856305

[26] MacKenzie K , Savage SAH , Birchall MA . Processes and outcomes of head and neck cancer patients from geographically disparate regions of the UK. A comparison of Scottish and English cohorts. Eur J Surg Oncol. 2009;35:1113‐1118. 19406610 10.1016/j.ejso.2009.04.001

[27] NDRS . NHS digital/NDRS ‐ cancer data get data out ‐ head and neck. 2021.

[28] Khalil D , Corsten MJ , Holland M , Balram A , McDonald JT , Johnson‐Obaseki S . Does socioeconomic status affect stage at presentation for larynx cancer in Canada's universal health care system? Otolaryngol Head Neck Surg. 2019;160:488‐493. 30200820 10.1177/0194599818798626

[29] Scottish Government Poverty and Income Inequality Statistics. 2023.

[30] Luryi AL , Yarbrough WG , Niccolai LM , et al. Public awareness of head and neck cancers: a cross‐sectional survey. JAMA Otolaryngol Head Neck Surg. 2014;140:639‐646. 24902640 10.1001/jamaoto.2014.867

[31] Sheikh I , Ogden J . The role of knowledge and beliefs in help seeking behaviour for cancer: a quantitative and qualitative approach. Patient Educ Couns. 1998;35:35‐42. 9832895 10.1016/s0738-3991(98)00081-0

[32] Buzelli L , Williamson S , Gardner T , et al. Public perceptions of the NHS: a winter of discontent Public perceptions of the NHS: a winter of discontent. The Health Foundation. 2023;2.

[33] Donaghy E , Sweeney K , Henderson D , et al. Primary care transformation in Scotland: a qualitative evaluation of the views of patients. Br J Gen Pract. 2024;74:e702‐e708. 38228359 10.3399/BJGP.2023.0437 PMC11104515

[34] Mercer SW , Watt GCM . The inverse care law: clinical primary care encounters in deprived and affluent areas of Scotland. Ann Fam Med. 2007;5:503‐510. 18025487 10.1370/afm.778 PMC2094031

[35] Woodley N , Rogers ADG , Turnbull K , et al. Prognostic scores in laryngeal cancer. Eur Arch Otorhinolaryngol. 2022;279:3705‐3715. 35112153 10.1007/s00405-021-07233-2

[36] Scottish Crime and Justice Survey . Main Findings. Scottish government. 2019.

[37] National Records of Scotland . Drug‐Related Deaths in Scotland in 2022 – Report. National Records of Scotland. 2023.

[38] Yaniv D , Reuven Y , Lahav Y , et al. Supraglottic carcinoma in intravenous opioid drug abusers: a distinct disease with improved survival. Laryngoscope. 2021;131:E1190‐E1197. 32946621 10.1002/lary.29067

[39] Irawan C , Benbella LG , Rachman A , Mansjoer A . Factors that influence 2‐year progression‐free survival among head and neck cancer patients. J Epidemiol Glob Health. 2022;12:16‐24. 34846716 10.1007/s44197-021-00016-2 PMC8907350

[40] Sok M , Zavrl M , Greif B , Srpčič M . Objective assessment of WHO/ECOG performance status. Support Care Cancer. 2019;27:3793‐3798. 30721369 10.1007/s00520-018-4597-z

[41] Magnuson A , Bruinooge SS , Singh H , et al. Modernizing clinical trial eligibility criteria: recommendations of the ASCO‐friends of cancer research performance status work group. Clin Cancer Res. 2021;27:2424‐2429. 33563633 10.1158/1078-0432.CCR-20-3868 PMC8102305

[42] Han SH , Cho D , Mohammad R , et al. Use of the comprehensive geriatric assessment for the prediction of postoperative complications in elderly patients with head and neck cancer. Head Neck. 2022;44:672‐680. 34918845 10.1002/hed.26958

[43] Denissoff A , Huusko T , Ventelä S , Niemelä S , Routila J . Exposure to alcohol and overall survival in head and neck cancer: a regional cohort study. Head Neck. 2022;44:2109‐2117. 35713171 10.1002/hed.27125 PMC9545212

[44] Giraldi L , Leoncini E , Pastorino R , et al. Alcohol and cigarette consumption predict mortality in patients with head and neck cancer: a pooled analysis within the international head and neck cancer epidemiology (INHANCE) consortium. Ann Oncol. 2017;28:2843‐2851. 28945835 10.1093/annonc/mdx486 PMC5834132

[45] Van Imhoff LCR , Kranenburg GGJ , Macco S , et al. Prognostic value of continued smoking on survival and recurrence rates in patients with head and neck cancer: a systematic review. Head Neck. 2016;38:E2214‐E2220. 25900211 10.1002/hed.24082

[46] Sorgini A , Kim HAJ , Zeng PYF , et al. Analysis of the TCGA dataset reveals that subsites of laryngeal squamous cell carcinoma are molecularly distinct. Cancers (Basel). 2021;13:1‐13. 10.3390/cancers13010105 PMC7794818 33396315

[47] Ingarfield K , McMahon AD , Douglas CM , Savage SA , MacKenzie K , Conway DI . Inequality in the survival of patients with head and neck cancer in Scotland. Front Oncol. 2019;8:673. 30723696 10.3389/fonc.2018.00673 PMC6349751

[48] Scottish Government . The Scottish Health Survey the Glasgow Effect A National Statistics Publication for Scotland Topic Report. Scottish Government. 2010.

[49] Dyckhoff G , Warta R , Herold‐Mende C , et al. Chemoradiotherapy but Not Radiotherapy Alone for Larynx Preservation in T3. Considerations from a German observational cohort study. Cancers (Basel). 2021;13:3435. 34298650 10.3390/cancers13143435 PMC8306673

[50] Pignon JP , Bourhis J , Domenge C , Designé L . Chemotherapy added to locoregional treatment for head and neck squamous‐cell carcinoma: three meta‐analyses of updated individual data. Lancet. 2000;355:949‐955. 10768432

[51] Luo XN , Chen LS , Zhang SY , Lu ZM , Huang Y . Effectiveness of chemotherapy and radiotherapy for laryngeal preservation in advanced laryngeal cancer: a meta‐analysis and systematic review. Radiol Med. 2015;120:1153‐1169. 25981380 10.1007/s11547-015-0547-8

[52] Lacas B , Carmel A , Landais C , et al. Meta‐analysis of chemotherapy in head and neck cancer (MACH‐NC): an update on 107 randomized trials and 19,805 patients, on behalf of MACH‐NC group. Radiother Oncol. 2021;156:281‐293. 33515668 10.1016/j.radonc.2021.01.013 PMC8386522

